# Management of Left Atrial Tachyrhythms in the Setting of HFpEF with Pulsed-Field Ablation: Treating Fire with Water?

**DOI:** 10.3390/therapeutics1010006

**Published:** 2024-09-23

**Authors:** Tyler Chinedu Chinyere, Ikeotunye Royal Chinyere

**Affiliations:** 1 College of Medicine—Phoenix, University of Arizona, Phoenix, AZ 85004, USA; 2 Sarver Heart Center, University of Arizona, Tucson, AZ 85724, USA; 3 Banner University Medicine, Banner University Medical Center—Tucson, Banner Health, Tucson, AZ 85719, USA

**Keywords:** electrophysiology, chronic fibrosis, heart failure, atrial fibrillation, nonthermal, catheter

## Abstract

Atrial fibrillation (AF) in the setting of heart failure (HF) with preserved ejection fraction (HFpEF) is a prevalent comorbidity and is enabled by adverse left atrial (LA) remodeling, dilation, and scar tissue formation. These changes are facilitated by poor left ventricular compliance. A growing body of clinical evidence and medical guidelines suggest that managing atrial tachyrhythms with catheter ablation (CA) is paramount to treating concomitant HF. This recommendation is complicated in that thermal CA modalities, namely radiofrequency ablation and cryoablation, are both therapeutic via inducing additional scar tissue. AF treatment with thermal CA may compound the atrial scar burden for patients who already have extensive scars secondary to HFpEF. Therefore, thermal CA could act as “gasoline” to the slowly burning “fire” within the LA, increasing the rate of AF recurrence. Pulsed-field ablation (PFA), which utilizes high-voltage irreversible electroporation, is a non-thermal CA technique that is capable of disrupting reentrant microcircuits and arrhythmogenic foci without inducing significant scar burden. PFA has the potential to mitigate the strong fibrosis response to thermal CA that predisposes to AF by serving as “water” rather than “gasoline”. Thus, PFA may increase the efficacy and durability of CA for AF in HFpEF, and subsequently, may decrease the risk of procedural complications from repeat CAs. In this article, we provide a summary of the clinical concepts underlying HFpEF and AF and then summarize the data to date on the potential of PFA being a superior CA technique for AF in the setting of comorbid HFpEF.

## 1. Introduction

### 1.1. Heart Failure

Heart failure (HF) is a clinical condition that affects sixty-four million people worldwide [1]. In the United States alone, approximately six million people over the age of twenty have HF [2]. The prevalence of HF in the United States is projected to increase 46% between the year 2012 and the year 2030. The increase in prevalence is the result of the increasing lifespan of individuals and improved management of chronic diseases [3]. Additionally, an average of 550,000 new cases will be diagnosed each year in the United States [4].

Treatment options for HF at all stages and the subsequent complications of HF have improved. As a result, approximately 9% of Americans died from HF in the year 2023 [2]. HF is managed with lifestyle optimization, medications, devices, and/or surgery. Lifestyle changes are the first therapeutic opportunities, as they address the root cause of adverse left ventricular (LV) remodeling. These changes are focused on regular exercise, healthy stress management, and most importantly, adequate nutrition, as exemplified in diets such as the Mediterranean diet [5]. Additionally, minimizing sedentary habits and eliminating hazardous exposures, such as tobacco and alcohol, also play a major role in preventing HF. Lifestyle changes are often coupled with medications to manage chronic conditions. Different classes of pharmaceutical agents can be used to manage cardiovascular disease risk factors, such as angiotensin-converting enzyme inhibitors, beta-adrenergic antagonists, and sodium-glucose cotransporter-2 inhibitors.

Devices are the third option for HF management, particularly with the complication of cardiogenic shock. These devices include cardiac contractility modulation [6], cardiac resynchronization therapy, and intra-aortic balloon pumps, as well as advanced mechanical circulatory devices for acutely depressed heart function, such as Impella^®^ heart pumps, extracorporeal membrane oxygenation, and HeartMate and BiVACOR^®^ ventricular assist devices. Cardiac contractility modulation operates by sending non-conductive electrical impulses to the heart, stimulating the cardiac tissue to contract and pump blood. Impella^®^ is an intravascular device that is temporarily placed into the ventricular cavity and pumps blood across the respective semilunar valve into the appropriate great artery via centrifugal flow, increasing the apparent cardiac output while simultaneously decreasing cardiac work. Extracorporeal membrane oxygenation (ECMO) is a treatment option that serves individuals with pulmonary complications, often in addition to cardiogenic shock. Blood rich in carbon dioxide is drawn out of the caval bodies and/or right atrium into a bladder box/bubble collector by a centrifugal pump. The pump then pushes the blood into a series of chambers (determined by sweep speed) where carbon dioxide is removed and oxygenation is performed via a gas blender and oxygenator. The temperature of the blood is controlled by a heat exchanger. Finally, the blood is returned to the body, either via the venous system (termed VV-ECMO) or arterial system (termed VA-ECMO; necessitates an additional device for left ventricular venting unless paired with a TandemHeart device). Finally, ventricular assist devices, in a unilateral or bilateral approach, may be used as a bridge to transplantation or as destination therapy.

The fourth and final modality for treating HF is surgery. Surgical options include valvular repairs or replacements for secondary heart failure; however, in the absence of valvular disease, the only curative treatment for HF is orthotopic heart transplantation. Transplantation carries its own risks and potential complications. Due to their non-invasive nature and low cost, medication and lifestyle changes are the preferred method of treatment. However, as the average age of HF patients continues to rise, advanced HF therapies will continue to increase in prevalence out of necessity. Regenerative medicine approaches continue to increase, as well as humanized organ transplants from swine, however, none have proven clinically worthwhile to date.

### 1.2. Atrial Fibrillation

Atrial fibrillation (AF) is the most common type of arrhythmia and affects forty million individuals globally [7]. The prevalence is expected to continue to increase in upcoming years due to prolonged life expectancies. AF was the cause of over 287,000 deaths internationally in 2017 [8]. In the United States, AF has a prevalence of six million individuals and caused or contributed to the deaths of 276,373 individuals from the year 2011 to 2018 [9]. The United States Centers for Disease Control and Prevention estimates the prevalence of AF will double by the year 2030 [7]. Managing AF includes rate- and rhythm-control medications, direct-current cardioversions, catheter ablations potentially with implantable devices, or surgical ablations.

Individuals being treated with medications can take one class of drugs or a combination of medications from distinct classes. Rate-control medications consist of beta-adrenergic antagonists, non-dihydropyridine calcium channel blockers, and occasionally, digoxin. Rhythm-control medications include sodium and potassium channel blockers. Cardioversions can be performed via direct-current electrical shock or pharmacologic means. With electrical cardioversion, two transcutaneous pads are first placed on an individual’s chest, or two paddles are placed within the mediastinum during open surgery. Then, a short pulse of high-voltage current is sent through the heart. The goal of the shock is to spontaneously depolarize all of the cells of the heart and allow the intrinsic conduction system, which depolarizes spontaneously at the fastest rate of all of the cardiac cells under normal circumstances, to capture the heart, resulting in sinus rhythm.

### 1.3. Ablation Techniques for Atrial Fibrillation

Catheter ablation techniques can be thermal in mechanism or non-thermal. Thermal ablations have long been the cornerstone treatment for AF refractory to pharmacologic suppression. Thermal catheter ablation procedures begin with inserting the catheter into a vascular (typically venous) access point in the groin and/or arm. The catheter then advances in the venous system until it enters the right atrium through the inferior vena cava. Once in the right atrium, the foramen ovale is used to bypass the right ventricle and lungs, entering the left atrium (LA). Once in the LA, the ablation catheter projects heat via radiofrequency energy or necrosis-inducing cold via circulating liquid nitrogen for the destruction of cardiac tissue that enables or propagates reentrant microcircuits and arrhythmogenic foci. It should be noted that arterial access and subxiphoid epicardial access are also utilized depending on substrate complexity and transmurality. Meta-analyses conducted on thermal catheter ablation found that a single ablation session of long-standing persistent AF, without pharmacologic substrate suppression, yielded only a 57% success rate [10], defined as no recurrent AF 12 months post-ablation. Furthermore, in the same study, the authors reported that after two or more ablations, the success rate then increased to 71%.

Surgical ablations, which are most commonly performed as one of the many MAZE, hybrid MAZE, or “cut-and-sew” MAZE procedures, require open heart surgery. In this form of ablation, a scalpel can be used to physically disconnect portions of the LA, pulmonary venous inlets, superior vena cava, and/or the right atrium. The disconnected tissue is then re-connected using a suture [11]. Ablation lines can also be created using intracardiac cryoablation or radiofrequency ablation. Due to electrical currents being unable to travel through the resultant scar tissue, abnormal electrical impulses from ectopic cardiac tissue are prevented from disrupting the regular heartbeat. The MAZE-III procedure has data supporting up to 90% of patients free of AF after one year [12]. Therefore, surgical ablations likely have increased efficacy compared to the 65–82% success rate reported for thermal catheter ablation.

### 1.4. Correlation between Heart Failure and Atrial Fibrillation

**Figure 1.**
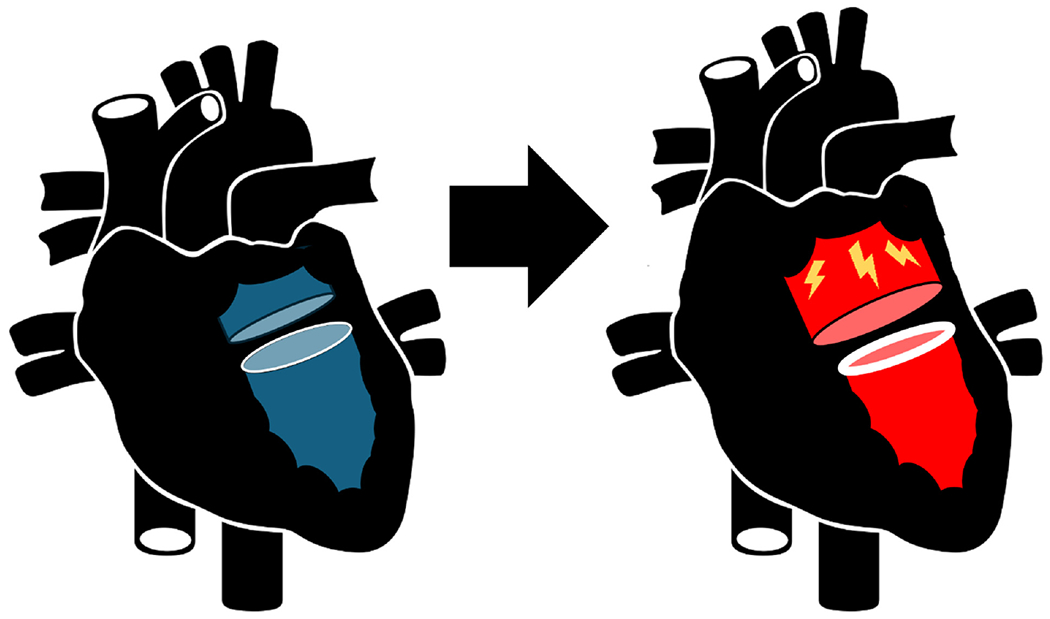
Pathological relationship between HFpEF and atrial fibrillation. Heart failure with preserved ejection fraction is a non-ischemic cardiomyopathy that is characterized by impaired diastolic function. This abnormally high ventricular pressure increases the incidence of left atrial pressure overload, inducing compensatory structural changes, including chamber dilation and subsequent myofibroblast proliferation for fibrosis. These changes predispose to atrial fibrillation induction.

Although both AF and heart failure with preserved ejection fraction (HFpEF) are distinct chronic conditions, a positive correlation exists between the two diseases (Figure 1). This is due to the fact that HFpEF can cause LA remodeling, irreversible dilation, and fibrosis, which is then capable of begetting AF. Moreover, the two comorbid conditions share risk factors, such as hypertension, acquired diabetes mellitus, and coronary artery disease. In two distinct studies [13,14], it was found that the prevalence of comorbid AF in the setting of HFpEF ranges from 33% to 65%.

The present review article outlines the underlying pathophysiology of AF in the setting of HFpEF. Moreover, we will evaluate the differences in outcomes between thermal catheter ablation and pulsed-field ablation (PFA) as treatments for AF, and also discuss the advantages and pitfalls of PFA. Finally, we will describe the data that support the theoretical advantage of PFA over thermal catheter ablation in patients with comorbid HFpEF and AF.

## 2. The Fire of HFpEF and AF

### 2.1. Heart Failure with Preserved Ejection Fraction

HF is clinically defined as any functional or structural impairment of the heart that prevents the ventricles from receiving or ejecting sufficient blood volume to meet the body’s perfusion needs [15]. Physical symptoms of HF include dyspnea while performing daily activities, orthopnea, fatigue, edema in the lower extremities, reduced exercise tolerance, and a persistent non-productive cough [16]. Clinical signs of HF include tachycardia, right ventricular heaving, elevated jugular venous pressure, pulsus or electrical alternans [17], and a displaced cardiac apex [16]. Among a variety of techniques, HF can be categorized based on the LV ejection fraction (EF). EF is the percentage of blood in the heart that is ejected with each heartbeat. Non-invasive cross-sectional imaging is the primary method of quantifying EF, accomplished primarily via echocardiography, computed tomography, or magnetic resonance (MR) imaging. Direct visualization of the LV cavity with contrast during cardiac catheterization (ventriculogram) can also be utilized to quantify EF, however, transthoracic ultrasound is the favored method due to speed, convenience, and highly reproducible methods.

Mathematically, EF is calculated by dividing the LV stroke volume—the amount of blood pumped out of the LV, as measured by end-diastolic volume minus end-systolic volume—by the LV end-diastolic volume, and then multiplying the fraction by 100. The four classes of HF include heart failure with reduced ejection fraction (HFrEF), heart failure with recovered ejection fraction (HFrecEF), heart failure with mid-range ejection fraction (HFmrEF), and HFpEF. HFrEF is defined as having an LVEF of less than or equal to 40%. HFmrEF is defined as having an LVEF of 41–49%. HFrecEF is defined as having an LVEF of greater than 40% after previously having an LVEF of less than or equal to 40%. Lastly, HFpEF is defined as having an LVEF of greater than or equal to 50%.

This article will focus specifically on HFpEF. HFpEF is caused by the inability of the LV to achieve complete relaxation and remains partly constricted. Clinically, HFpEF is a relatively new entity and as such, presents significant challenges with regard to diagnostic certainty and pharmacologic management. The demographic of patients who suffer from HFpEF rarely overlaps with those who suffer from HFrEF, in that the former cohort tends to be female, lack obstructive coronary disease, and may be advanced in age consequently with a higher relative burden of comorbidities.

Multiple molecular theories underlie the clinical phenomenon of HFpEF, though the most prominent is the concept of titinopathy. Titin is the largest protein of the sarcomere and is also the largest protein in the entire body. Titin is the primary driver of the cardiomyocyte’s diastolic spring and has been studied extensively with regard to HFpEF [18]. Impaired relaxation of the LV leads to the incomplete filling of the LV with blood; however, the systolic function of the LV is not impaired. A reduced end-diastolic volume with normal systolic function preserves the numerical fraction for EF but leads to a lower cardiac output that is not able to meet the body’s metabolic demands [19]. Moreover, the excessive stiffness of the LV causes a pressure imbalance in the serial cardiac chambers, which in turn causes the LA to dilate and accumulate scar tissue [20].

LA fibrosis is significant because it has a positive correlation with the likelihood of adverse events such as thromboembolism [21]. Additionally, fibrosis of the LA can lead to tachyrhythms; therefore, individuals with HFpEF are frequently diagnosed with AF. It is well known that LA is a major target for treating AF [21]. Risk factors for HFpEF are increased age, AF, renal disease, hypertension, and acquired diabetes mellitus. Mortality rates of patients with HFpEF are 8% for individuals 70 and younger, and 12% for individuals 71 and older. Interestingly, 17% of individuals with HFpEF die from a comorbid condition [17].

### 2.2. Atrial Fibrillation in the Setting of HFpEF

AF is the most common type of arrhythmia in the world, affecting more than forty million individuals worldwide [7]. The cellular mechanism of AF begins when previously contractile atrial cardiomyocytes, typically located in the LA near the pulmonary vein inlets, become capable of self-excitation and depolarization, allowing them to initiate an action potential in lieu of the intrinsic cardiac pacemaker cells. Once a few of the now-autorhythmic cardiomyocytes achieve depolarization, they can facilitate a depolarization wavefront across the atrial myocardium [22]. Many of these wavefronts can co-exist spontaneously due to the spatial separation of the conductive tissue and the relatively short refractory period. These wavefronts are often depicted as spiral wavelets, triggered by unique foci of ectopic electrical activity, and occasionally merge into seemingly synchronous atrial electrical activity. The uncoordinated electrical activity leads the atria to mechanically quiver rather than uniformly contract, leading to the elevated thromboembolic risk associated with AF [23]. Clinically, this phenomenon can be observed on a 12-lead surface electrocardiogram by the absence of discrete P-waves and irregular RR intervals [23].

AF can be classified into four different stages [24,25]: paroxysmal (stage 3A), persistent (stage 3B), long-standing persistent (stable 3C), and permanent (stage 4). Rhythm control is one of the two major dogmas utilized to manage AF. It involves the use of antiarrhythmic medications (predominately class 1 and class 3 agents), cardioversions, catheter ablations, or surgical ablations to induce sinus rhythm [26]. The other option for AF treatment is rate control. The goal of rate control is to slow the ventricular rate without re-establishing sinus rhythm. This is accomplished via class 2 and class 4 agents [27].

Increasing clinical evidence and medical guidelines suggest that managing tachyrhythms with catheter ablation is paramount to treating AF concomitant to HF [28,29]. Catheter ablation of AF in patients with HFrEF has now attained a Class 1 indication based on contemporary randomized trial data that have demonstrated the superiority of catheter ablation over drug therapy for rhythm control [30,31]. It could be postulated that HFpEF is not far behind.

Due to the use of thermal energy as the mechanism of action, cardiac fibrosis is often induced by radiofrequency and cryoablation. In patients suffering from AF without HFpEF, this treatment is a reasonable option and has a variable success rate in maintaining sinus rhythm, previously documented as high as 56% two years post-thermal ablation [29]. Although thermal ablation has long been the only option for catheter-based rhythm management for AF, extra consideration should now be given due to the fact that patients suffering from HFpEF complicated by AF may benefit from a catheter ablation modality that does not induce additional macroscopic scar tissue. HFpEF patients have a greater fibrosis burden in the LA, secondary to the increased pressure exposure from the LV process. Therefore, treatment of AF with a thermal ablation technique may compound the degree of scar tissue burden for patients with extensive LA scar tissue. This can be thought of as adding “gasoline” to the slowly burning “fire” within the LA.

### 2.3. Pulsed-Field Ablation

PFA is a “non-thermal” ablation technique (though microscopic temperature changes have been observed, depending on the pulse frequency and voltage) that is gaining international attention for the treatment of AF. The FARAPULSE^™^ system has been increasingly appearing in invasive cardiac electrophysiology labs across the United States with recent Food and Drug Administration approval, although multiple commercial PFA systems are available worldwide. PFA utilizes irreversible electroporation to facilitate cell death in membranous cells and intracellular organelles. The electrical impulses act by creating pores in the cell membrane, thus destabilizing the cell membrane and inducing cell death via apoptosis or necrosis [32,33].

PFA’s mechanism of action specifically targets phospholipid bilayer membranes. By causing intra-membrane current flow via high-voltage electrical pulses, lethal membrane damage is induced. This damage theoretically occurs to all membranes within the high-voltage electric field, though susceptibilities to PFA vary by tissue type and the exact PFA parameters can be modulated. PFA electrical pulses can be tuned for the impulse length, and set to monophasic or biphasic in addition to monopolar or bipolar. Multiple unique studies [34–36] have shown that short biphasic pulses have greater success rates compared to monophasic impulses; however, there is no universal protocol guiding operators at this time. Similarly, the exact voltage selection is made by the operator [34]. Despite there being no publicly available universal protocol, most publicly available documented PFA procedures utilize approximately 10–90 pulses during each session, with each pulse lasting 100 microseconds, along with an electric field of roughly 500–3000 V/cm, and a frequency of 1–10 Hz [34]. Although there are limited amounts of data on the effectiveness of PFA for comorbid AF in the setting of HFpEF, the theoretical advantages of a non-thermal catheter ablation technique may prove that PFA is superior compared to radiofrequency and cryoballoon catheter ablation (Table 1). Thermal ablation induces additional atrial fibrosis and increases the risk of subsequent AF.

**Figure 2.**
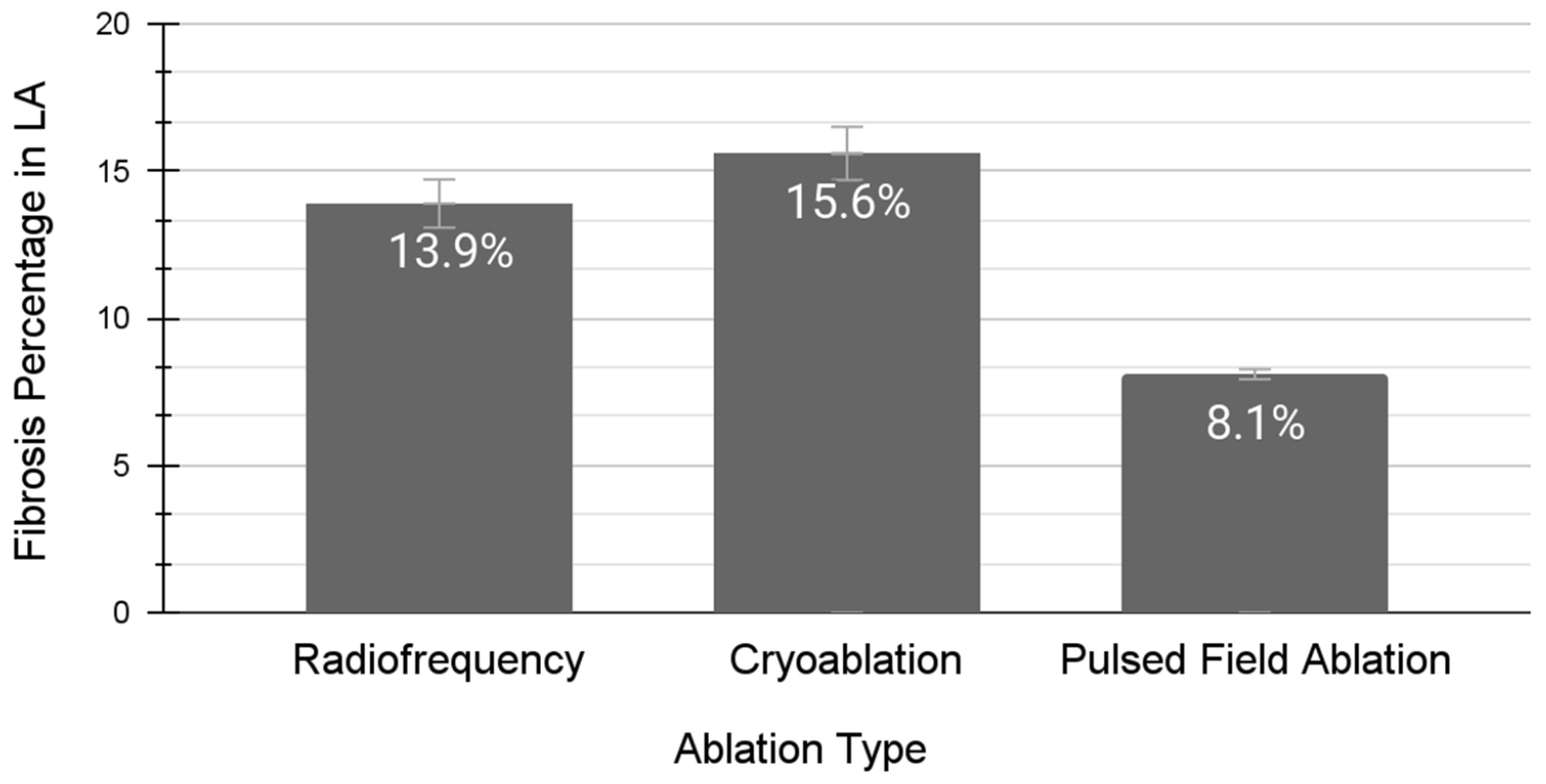
Post-catheter ablation burden of left atrial fibrosis for atrial fibrillation. The percentage of scar tissue formation in the left atrium (LA) after treatment with either catheter radiofrequency ablation [44], cryoablation [45], or pulsed-field ablation [46]. LA scar burden was estimated via late-gadolinium enhancement on cardiac magnetic resonance imaging for all three distinct studies depicted in Figure 1. The pulsed-field ablation and radiofrequency ablation studies measured the LA scar burden three months post-procedure. The cryoablation study measured the LA scar burden 362 days post-procedure. Mean ± standard deviation: radiofrequency (0.139 ± 0.059, *n* = 70), cryoablation (0.156 ± 0.058, *n* = 102), and pulsed-field ablation (0.081 ± 0.021, *n* = 10).

The first and primary advantage of PFA over thermal catheter ablation lies in its improved profile of procedural risks and side effects. Due to the non-thermal mechanism of PFA, the risks of thermal complications, such as perforation, fistula formation, and pulmonary vein stenosis, are essentially mitigated [37–39]. The second advantage of PFA is that electrical impulses are unaffected by adjacent high-velocity blood vessels, unlike in thermal ablation where the sink effect must be accounted for [40]. Blood vessels can decrease (in the setting of radiofrequency ablation) or increase (in the setting of cryoablation) the temperature of surrounding structures. These blood vessels can act as a buffering system and prevent the cardiac tissue from reaching the desired temperature [41]. This sink effect is mitigated with PFA, although an electro de contact/distance effect has been described. Another advantage of PFA is that it does not promote the same degree of scar tissue formation when compared to thermal ablation techniques (Figure 2) [42]. Given that the LA in individuals with HFpEF already has increased dilation and fibrosis, PFA should not add significant LA fibrosis burden, which theoretically should prevent additional risks of major adverse cardiovascular events [43–46].

Though numerous potential advantages exist, PFA certainly has potential pitfalls. First, and foremost, the novelty of PFA limits our knowledge about the long-term outcomes and side effects from the procedure. Although some specific procedural risks and complications are decreased with PFA, other risks, such as unintentional arterial vasoconstriction via smooth muscle damage [36], and the possibility of injury to surrounding nervous tissue [39], still exist. Iatrogenic coronary vasoconstriction is actively mitigated during PFA procedures via intracoronary nitroglycerin infusion, which inhibits contraction should smooth muscle damage occur. And although nervous tissue damage has been documented with clinical PFA application [39], no permanent phrenic nerve palsies have been documented to date. No data exist to quantify the clinical significance (if any) or extent of injury to the dense network of autonomic nervous tissue that innervates the cardiac system. These limitations also serve as future research directions, namely (1) consolidation of long-term outcomes data with PFA for the various forms of atrial tachyrhythms, (2) a clinical trial or post hoc analysis comparing thermal versus non-thermal catheter ablation outcomes for HFpEF patients, and finally, (3) a cardiac MR-guided or post-mortem analysis regarding the degree of left atrial fibrosis post thermal versus non-thermal catheter ablation.

With regard to the limitations of this proposed PFA application, it is important to note that AF is not exclusively an LA process. Secondly, no long-term data on the efficacy of PFA in the treatment of various forms of AF exist. Hence, the effect on HF (whether with preserved or reduced EF) is not known, although it has recently been adapted into the 2023 ACC/AHA/ACCP/HRS guidelines for the diagnosis and management of atrial fibrillation to treat HFrEF (class 1 recommendation) as well as HFpEF (class 2a recommendation) concomitant with AF with catheter ablation with regard to superior rhythm control compared to drug therapy. Although, in theory, the amount of fibrosis induced by PFA is reduced comparable to current thermal catheter and surgical ablation techniques, the data from initial randomized trials have only demonstrated superiority in terms of preventable complications. Additional studies with regard to PFA efficacy on various subtypes of AF as well as PFA-induced atrial remodeling/fibrosis are still necessary. Moreover, clinical measures such as the blanking period are still being established for PFA [47].

In summary, PFA provides a way to treat pharmacologically resistant AF, reducing concerns for worsening LA scar burden or inadvertently creating iatrogenic conditions for the patient. Additionally, PFA may decrease the rate of ablation failure for AF, as well as the number of procedures needed for recurrent AF. Ultimately, PFA has the potential to act as “water” to quench the slowly burning “fire” of the LA that is predisposed to AF. The invasive electrophysiologist armamentarium is bolstered with the opportunity to deploy both non-thermal and thermal ablation technologies. Future studies should aim to compare outcomes in this unique patient subset of AF with HFpEF, and ultimately inform medical guidelines on a superior ablation modality.

## 3. Conclusions

PFA has the potential to be a superior alternative to treat AF in the setting of HFpEF when compared to thermal ablation. This theoretical advantage is based on the evidence that thermal ablation induces fibrosis, which can lead to recurrent AF, serving as “gasoline” to the “fire” of LA-driven AF. In contrast, PFA utilizes non-thermal energy and, therefore, has a diminished likelihood of recurrent AF and elevated risk factors, serving as “water” to the “fire”. Although PFA holds exceptional promise for managing AF in the setting of HFpEF, dedicated clinical studies are needed to provide additional evidence to guide its use.

## Figures and Tables

**Table 1.** Comparison of thermal catheter ablation and pulsed-field catheter ablation. A summary of the pertinent factors that differentiate thermal catheter ablation and pulsed-field catheter ablation technologies, patient factors, and durability.

	Thermal Ablation	Pulsed-Field Ablation
Mechanism of Action	Necrosis of all local cells along with destruction of extracellular matrix and acellular connective tissue	Irreversible electroporation of membrane-bound cells including cardiomyocytes without disruption of extracellular matrix and acellular connective tissue
Specific Contraindications	Intracardiac locations at a high risk for fistula formation	Higher than average propensity to vasospasm coronary arteries
Blanking Period	3 months	Estimated to be 1 month presently
Long-term Durability	High, potentially curative after serial ablations	Unknown presently, potentially high

## Data Availability

All utilized data is contained within the manuscript.
